# From Seeing to Healing: The Clinical Potential of Radiotracers in Pediatric Neuro-Oncology

**DOI:** 10.3390/cancers17121905

**Published:** 2025-06-07

**Authors:** Bojana Bogdanović, Christopher Montemagno

**Affiliations:** 1 Grenoble Alpes University, INSERM, LRB, 38000 Grenoble, France; bojana.bogdanovic@univ-grenoble-alpes.fr; 2 Département de Biologie Médicale, Centre Scientifique de Monaco, 98000 Monaco City, Monaco

**Keywords:** pediatric brain tumors, molecular imaging, radioligand therapy, radiopharmaceuticals, theranostics

## Abstract

Pediatric central nervous system (CNS) tumors, including gliomas, medulloblastomas, and diffuse midline gliomas (previously diffuse intrinsic pontine gliomas), remain a major clinical challenge due to their complex biology, limited treatment effectiveness, and generally poor prognosis. Standard treatments are often aggressive and associated with substantial toxicity, particularly in advanced stages. This review highlights recent developments in radiopharmaceuticals for molecular imaging and targeted radiotherapy. A comprehensive literature analysis was conducted, focusing on radiotracers with clinical relevance in pediatric neuro-oncology, including metabolic, peptide receptor-based, and antibody-based agents. Radiopharmaceuticals such as ^18^F-FLT, ^64^CuCl_2_, and 1-L-18F-FETrp have improved the ability to monitor tumor biology, proliferation, and treatment response, aiding in diagnosis at an early stage, assessment of tumor behavior, and detection of recurrence or progression. Additionally, peptide receptor-based radiotracers, such as ^68^Ga-DOTATATE and ^177^Lu-DOTATATE, are already used for both diagnostic purposes and targeted radiotherapy, particularly in neuroblastomas and gliomas. Antibody-based radiotracers like ^131^I-omburtamab, targeting B7-H3, are emerging as promising tools for addressing difficult-to-treat tumors such as diffuse midline glioma. Collectively, these advances provide new hope for children afflicted by these devastating malignancies, offering promising solutions for more specific and precise diagnosis and, additionally, for more effective, personalized, and less toxic tumor therapies.

## Simple Summary

Pediatric central nervous system tumors, including gliomas, medulloblastomas, and diffuse midline gliomas, are difficult to treat and often involve harsh therapies with significant side effects. Recent advances in molecular imaging and targeted therapies provide more personalized and less toxic treatment options. This review highlights progress in radiopharmaceuticals and imaging agents, such as metabolic tracers and peptide receptor-based radiotracers, which improve tumor monitoring and treatment precision. Antibody-based radiotracers also show promise for these challenging tumors. Combining diagnostic imaging with targeted therapy offers the potential to reduce side effects and improve outcomes for children with these complex tumors.

## 1. Introduction

Pediatric central nervous system (CNS) tumors, particularly brain tumors, are the most common solid tumors in children, accounting for approximately 20% of all childhood cancers and representing the second most frequent pediatric malignancy [1,2]. Astrocytic tumors and other glioma subtypes—including unspecified gliomas—together with medulloblastoma are the most common pediatric brain tumor entities reported in population-based registries [1,2]. Despite significant progress in understanding their molecular underpinnings, pediatric brain tumors remain the leading cause of cancer-related death in children [1,2,3].

Characterized by their heterogeneity and frequent proximity to critical brain structures, pediatric brain tumors present significant therapeutic challenges [4]. For instance, diffuse midline glioma (DMG), classified as diffuse intrinsic pontine glioma (DIPG) prior to the 2021 WHO CNS tumor classification, has a dismal prognosis due to its inoperability, while low- and high-grade gliomas (L/HGG) and MBs are prone to recurrence and resistance to standard therapies [4,5,6]. Early and accurate diagnosis, along with the development of targeted therapies strategies, is crucial—especially in low- and middle-income countries, where data on incidence and mortality are often limited or unavailable [7].

Conventional anatomical imaging techniques, such as magnetic resonance imaging (MRI), are widely used and remain the gold standard for CNS tumor diagnosis and follow-up, while computed tomography (CT) remains essential in emergency care [8]. However, these methods have limitations, particularly in distinguishing tumor progression from treatment-related changes, such as pseudoprogression and radiation necrosis [9,10].

To overcome these challenges, functional imaging techniques like positron emission tomography (PET) and single-photon emission computed tomography (SPECT) are increasingly integrated into clinical workflows [11]. By using radiolabeled molecules or radiotracers, they provide non-invasive, high-resolution imaging of tumor metabolism, receptor expression, and molecular activity, offering more precise diagnosis and treatment planning [12]. Furthermore, the same radiotracers used for functional imaging can also be employed in targeted radiotherapy, a promising theranostic approach that combines diagnostic and therapeutic capabilities for personalized treatment strategies [13].

**Figure 1:**
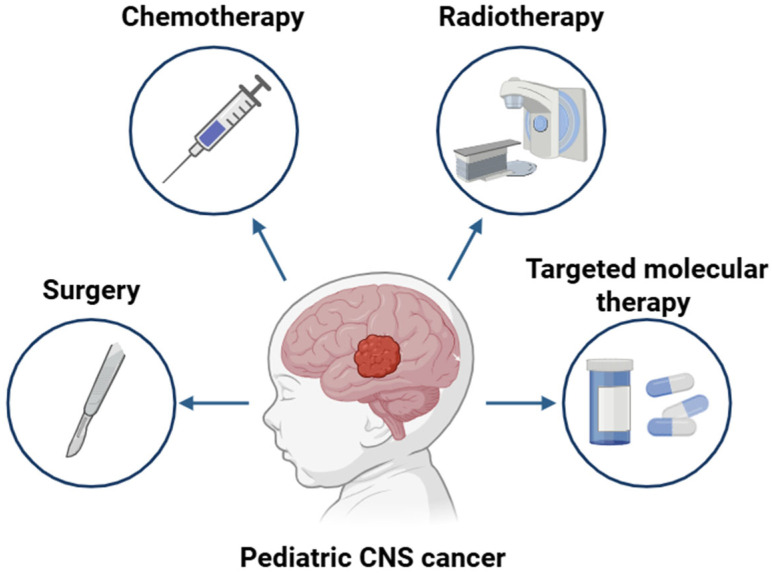
Multimodal treatment strategies for pediatric CNS cancer. Treatment strategies in pediatric neuro-oncology typically include surgery, chemotherapy, and radiation therapy, applied alone or in combination depending on tumor type, location, and patient-specific factors. Additionally, targeted molecular therapies, such as MAPK pathway inhibitors, have been approved for certain molecularly defined pediatric low-grade gliomas (LGGs), offering a more personalized and potentially less toxic treatment option [22]. This figure highlights the diversity of approaches and underscores the need for precise diagnostic and prognostic tools—such as PET and SPECT imaging—to guide personalized treatment planning and monitor response.

Given the complexity and variability of therapeutic strategies in pediatric brain tumors, functional imaging plays a crucial role in guiding clinical decisions. As illustrated in Figure 1, the choice of treatment, whether surgical, chemotherapeutic, radiotherapeutic, or targeted treatment-based, depends heavily on tumor characteristics, many of which can be more accurately assessed through advanced imaging techniques such as PET and SPECT.

As a result, the field has moved toward multimodal imaging strategies that integrate anatomical detail with functional information [14]. Among these, hybrid PET/MRI and PET/CT systems represent significant advancements, enhancing lesion characterization, treatment response assessment, and detection of recurrence [15]. Notably, compared to PET/CT, PET/MRI offers simultaneous acquisition of high-resolution metabolic and structural data within a single session. This results in superior anatomical correlation owing to MRI’s excellent soft-tissue contrast, alongside a substantial reduction in radiation exposure—an especially critical advantage in pediatric neuro-oncology, where patients frequently require multiple follow-up scans [16,17]. Additionally, MRI offers advanced sequences such as diffusion-weighted imaging (DWI), arterial spin labeling (ASL), and spectroscopy, which provide additional physiological and metabolic information about tumor cellularity, perfusion, and biochemical composition [18]. The integration of PET and MRI thereby enhances diagnostic accuracy and safety for long-term monitoring in children, reinforcing PET/MRI as an invaluable tool for personalized patient care.

Despite these advantages, the integration of PET and SPECT in pediatric neuro-oncology has lagged behind adult practice. Factors such as limited pediatric-specific data, the need for sedation, and concerns regarding radiation exposure have restricted widespread adoption [19]. However, these modalities are increasingly recognized for their roles in staging, monitoring disease progression, and providing targeted therapy in pediatric brain cancers [20,21].

## 2. Clinically Established Radiotracers in Pediatric Oncology: Applications and Limitations in CNS Tumors

In pediatric oncology, a limited number of radiotracers are routinely used in clinical practice to evaluate tumor metabolism, monitor treatment response, and guide biopsy or surgical planning. Among them, ^18^F-FDG (fluorodeoxyglucose), is one of the most widely used PET tracers in extracranial malignancies, taking advantage of the elevated glucose uptake seen in many malignant cells [21,23]. In pediatric brain tumors such as ependymoma, MB, and DMG, ^18^F-FDG has a selective utility for assessing metabolic activity and providing prognostic insight [24,25,26]. Its capacity to delineate metabolic heterogeneity also makes it a useful tool for guiding biopsies in unresectable tumors. However, its clinical utility is limited by high physiological uptake in normal gray matter, which can obscure low-grade lesions. Additionally, ^18^F-FDG accumulation in inflammatory or infectious sites can result in false-positive findings [27].

Another commonly used tracer is ^123^I-mIBG (^123^I-meta-iodobenzylguanidine), a SPECT agent targeting norepinephrine transporters (NETs) that are overexpressed in neuroendocrine tumors, most notably neuroblastoma (NB) [28,29,30]. While NB is not a primary CNS tumor, its origin in the sympathetic nervous system and potential for central nervous system metastasis make it occasionally relevant in pediatric neuro-oncology, especially in advanced or refractory cases [31]. CNS involvement has been reported in approximately 2–11% of cases, with higher rates observed in relapsed or stage IV disease cohorts, and is notably associated with poorer patient prognosis [32,33]. In such instances, ^123^I-mIBG scintigraphy can assist in detecting CNS involvement, but its role in brain metastasis detection remains limited—primarily due to poor blood–brain barrier penetration, the inherently low spatial resolution of SPECT, and the small or hemorrhagic nature of many metastatic foci that may not accumulate tracer reliably [31,34,35]. Furthermore, up to 10% of NB cases are mIBG-negative, further highlighting the need for improved and CNS-specific imaging agents [36].

## 3. Emerging Alternative Radiotracers in Pediatric Neuro-Oncology

**Figure 2:**
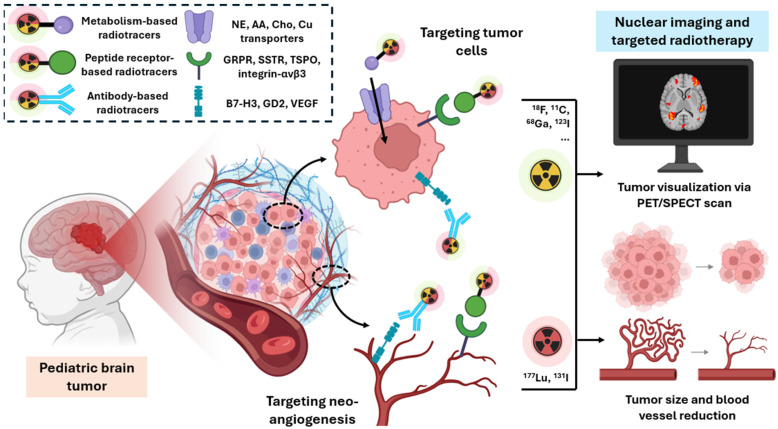
Overview of molecular targets identified in pediatric brain tumors and the corresponding radiotracers used for molecular imaging (PET/SPECT) or radioligand therapy. The figure highlights tracer types (metabolism-, peptide receptor-, and antibody-based), associated radionuclides (^18^F, ^68^Ga, ^123^I, and ^11^C for imaging—green radioelement; ^177^Lu and ^131^I for radioligand therapy—red radioelement), and their specific tumor-related targets, including receptors, tumor-associated antigens, and other relevant biomarkers located on tumor cells or the associated vasculature. Color coding reflects the functional use of the radiotracer (diagnostic vs. therapeutic). Both green and red tracers may target either tumor cells or tumor vasculature, depending on the specific agent.

The limitations of current standard radiotracers have driven the search for novel imaging agents that provide greater diagnostic precision, prognostic power, and therapeutic guidance. This review explores the latest advancements in metabolism-based, peptide receptor-based, and antibody-based radiotracers, summarized in Table 1, emphasizing their clinical status, evaluated tumor types, and decision-making utility to highlight their relevance for pediatric brain tumor imaging and therapy planning. The different types of radiotracers dedicated to the theranostic approach are presented in Figure 2.

**Table 1:** Summary of metabolism-based, peptide receptor-targeting, and antibody-based radiopharmaceuticals recently evaluated in pediatric neuro-oncology. This table provides an overview of each tracer’s mechanism, imaging modality, current clinical status, and evaluated tumor types, with a distinction between those widely used in clinical practice and those still under clinical or preclinical investigation. The final column summarizes key clinical applications, including diagnostic value, therapeutic decision-making, and response assessment. Abbreviations: cRIT (Convection-Enhanced Radioimmunotherapy); CTR1 (Copper Transporter 1); DAT (Diffuse Astrocytic Tumors); DMG (Diffuse Midline Glioma); ETMR (Embryonal Tumor with Multilayered Rosettes); GBM (Glioblastoma Multiforme); GD2 (Disialoganglioside 2); GRPR (Gastrin-Releasing Peptide Receptor); HGG (High-Grade Glioma); LAT1/LAT2 (L-type Amino Acid Transporter 1/2); LGG (Low-Grade Glioma); MB (Medulloblastoma); NB (Neuroblastoma); NET (Norepinephrine Transporter); NGGCT (Non-Germinomatous Germ Cell Tumor); OPG (Optic Pathway Glioma); PET (Positron Emission Tomography); PRRT (Peptide Receptor Radionuclide Therapy); SPECT (Single Photon Emission Computed Tomography); SSTR2/3/5 (Somatostatin Receptor Subtypes 2, 3, and 5); TK-1 (Thymidine Kinase 1); TSPO (Translocator Protein); VEGF (Vascular Endothelial Growth Factor).

Mechanism	Target	Imaging Modality	Targeting Compound	Clinical Status in Pediatric Neuro-Oncology	Evaluated Tumor Type	Clinical Utility	References
Metabolism-based	Amino-acid uptake via LAT1/LAT2	PET scan	^11^C-MET	In clinic (increasing)	DMG, HGG, LGG,	Recurrence detection; biopsy guidance; prognosis correlation	[37,38,39,40]
			^18^F-DOPA	In clinic (routine)	DAT, recurrent glioma, DMG, HGG, LGG, NB	H3K27M status distinguishment; survival prognostication; treatment response monitoring; pseudoprogression differentiation	[41,42,43,44,45,46,47,48]
			^18^F-FET	In clinic (routine)	DMG, astrocytomas, GBM, MB	High specificity for tumor vs. post-treatment changes; treatment plans modification; biopsy guidance	[49,50,51,52,53,54,55]
	Choline transport and phospholipid synthesis	PET scan	^18^F-Cho	Early clinical (Pilot studies)	astrocytic tumors, HGG, MB, NGGCT	Treatment response monitoring; biopsy guidance	[45,56,57]
	Norepinephrine uptake via NET	PET scan	^18^F-mFBG	Early clinical (Phase II)	NB	Staging; avoiding sedation; early treatment evaluation	[58,59,60,61,62,63,64,65]
			^124^I-mIBG	Early clinical (Pilot study)	NB	Improved lesion detection and coverage; superior to SPECT/CT; dosimetry planning	[66]
	DNA synthesis via TK-1	PET scan	^18^F-FLT	Early clinical (Phase II)	CNS tumors	Recurrence assessment; early treatment response monitoring	[67,68]
	Copper uptake via CTR1	PET scan	^64^CuCl_2_	Early clinical (Pilot study)	HGG, DMG	Necrosis alignment; theranostic potential	[69]
	Kynurenine pathway	PET scan	1-L-^18^F-FETrp	Preclinical study	MB (mouse model)	Potential diagnostic role	[70,71]
Peptide receptor-based	SSTR2	PET scan	^68^Ga-DOTATATE	In clinic (increasing, off-label use)	NB, CNS metastasis	Lesion detection improvement in SSTR2+ NB; PRRT candidate identification; CNS metastases detection	[72,73,74,75]
		SPECT scan	^177^Lu-DOTATATE	Early clinical (Phase II)	NB	PRRT in refractory SSTR2+ NB	[76,77,78,79,80,81]
	SSTR2/3/5	PET scan	^68^Ga-DOTANOC	Case reports	MB	SSTR+ metastasis detection; Treatment response monitoring	[82,83]
	GRPR	PET scan	^68^Ga-NOTA-Aca-BBN(7–14)	Early clinical	OPG	Surgical planning assistance	[84]
	TSPO	PET scan	^18^F-DPA-714	Preclinical studies	DMG (rat model)	Potential diagnostic role	[85]
	Integrin-αvβ3	SPECT scan	^99m^Tc-RAFT-RGD	Preclinical studies	MB (mouse model)	Potential diagnostic role	[86]
Antibody-based	B7-H3 (CD276)	PET scan, PRRT	^124^I-omburtamab	Early clinical (Phase I)	DMG, NB	Lesion detection; Dosimetry; PRRT candidate identification	[87,88,89,90]
		SPECT scan	^131^I-omburtamab	Early clinical (Phase I)	NB, MB, ependymoma, ETMR,	Dosimetry, cRIT in leptomeningeal disease	[91,92,93]
	GD2	PET scan	^89^Zr-dinutuximab	Preclinical studies	NB (mouse model)	Potential for patient stratification for anti-GD2 therapy	[94]
		PET scan	^64^Cu-dinutuximab beta	Early clinical (Pilot study)	NB	Patient stratification for anti-GD2 therapy	[95]
	VEGF	PET scan	^89^Zr-bevacizumab	Early clinical (Pilot study)	DMG	Reveals intratumoral heterogeneity; anti-VEGF therapy candidate selection	[96,97]

### 3.1. Molecular Imaging with Metabolism-Based Radiotracers

#### 3.1.1. C-MET

^11^C-MET (O-(^11^C)-methyl-L-methionine) is the most extensively studied amino acid-based PET tracer for brain tumor imaging [98]. Unlike other tracers that primarily target LAT1/LAT2 transporters, ^11^C-MET reflects both amino acid transport and protein synthesis, making it particularly sensitive for detecting tumor activity and progression [99,100]. This dual mechanism of uptake enhances its ability to visualize metabolically active tumor regions, making it an important tool for tumor diagnosis, recurrence detection, and prognostic assessment.

In pediatric DMG, ^11^C-MET PET has demonstrated high tumor uptake in both baseline (82%) and post-treatment scans (88%), though uptake in MRI-defined tumor regions was more limited (61%) [37]. While baseline intensity and uniformity did not predict survival, ^11^C-MET identified recurrence-prone areas in 100% of cases, suggesting strong potential in recurrence risk stratification. A larger study carried out on DMG patients confirmed ^11^C-MET PET’s prognostic role, showing that metabolic tumor volume (MTV), T/B ratio, and uptake uniformity correlated with shorter PFS and OS [38]. However, uptake patterns did not correlate with H3K27M mutation status, indicating limited ability to differentiate molecular subtypes.

In pediatric high-grade gliomas (HGG), where distinguishing recurrence from pseudo-progression is clinically critical, ^11^C-MET PET outperformed MRI in sensitivity, specificity, and interobserver agreement (100% vs. 50%) [39]. For low-grade gliomas (LGG), it showed high sensitivity in both new (93%) and previously treated (96%) cases, with post-therapy reductions in T/B ratios reflecting treatment response [40].

Despite these strengths, ^11^C-MET’s short half-life (~20 min) requires on-site cyclotron production, limiting accessibility. Consequently, many centers now prefer ^18^F-labeled alternatives like ^18^F-FET and ^18^F-DOPA, especially in Europe [99,100]. Nonetheless, ^11^C-MET remains a benchmark tracer, offering crucial metabolic insights when conventional imaging is inconclusive.

#### 3.1.2. ^18^F-DOPA

^18^F-DOPA (^18^F-dihydroxyphenylalanine) is an amino acid-based radiotracer originally designed for imaging the dopaminergic system [101]. Its high tumor-cell uptake and low background in normal brain tissue have made it a particularly valuable tool in neuro-oncology, particularly for distinguishing tumor progression from pseudoprogression and providing prognostic data [102,103]. As such, ^18^F-DOPA PET is increasingly integrated into clinical practice for the diagnosis, treatment planning, and therapeutic monitoring of pediatric CNS tumors.

In pediatric diffuse astrocytic tumors (DAT), PET-derived tumor-to-striatum (T/S) and tumor-to-normal (T/N) ratios independently predicted PFS, even when advanced MRI techniques like DWI and ASL were used concurrently [41]. PET/MRI integration further enhanced diagnostic accuracy, emphasizing the synergy of multimodal imaging.

^18^F-DOPA PET/MRI has demonstrated notable utility in monitoring treatment responses, particularly in pediatric recurrent gliomas undergoing bevacizumab therapy. Changes in metabolic tumor volume (MTV) measured via PET showed superior correlation with clinical outcomes compared to conventional measures like standardized uptake values (SUVmax) and tumor-to-brain (T/B) ratios, positioning PET as an effective early response biomarker [42].

Additionally, ^18^F-DOPA PET uniquely differentiated molecular subtypes of pediatric diffuse midline gliomas (DMG), specifically distinguishing H3K27M-mutant from wild-type DMGs [43]. Although MRI techniques successfully identified tumor grades, only PET-derived T/S ratios reliably separated these molecular subgroups, aiding personalized therapeutic decisions.

In DMG, higher baseline ^18^F-DOPA uptake (T/S ratio > 1) strongly correlated with poorer survival outcomes (≤12 months), highlighting aggressive tumor regions resistant to standard treatments [44]. Moreover, hybrid PET/MRI effectively differentiates true tumor progression from pseudoprogression across multiple pediatric brain tumors, including high-grade astrocytomas (H3.3 and H3F3A K27M mutations), DMGs, and LGGs, significantly influencing clinical decisions regarding treatment and response assessment post-radiotherapy or chemotherapy [45].

Interim ^18^F-DOPA PET imaging also provided crucial prognostic information in pediatric NB, with parameters such as whole-body metabolic burden and combined FDG/DOPA SUVmax ratios effectively predicting overall survival (OS) and PFS, thus facilitating early risk stratification during ongoing therapy [46].

Recent advancements in automated and semi-automated analysis methods for ^18^F-DOPA PET in pediatric gliomas have improved reproducibility, with automated T/B and T/S ratio calculations reliably predicting tumor grade and outcomes in pediatric gliomas [47,48]. These developments enhance the practicality of ^18^F-DOPA PET in clinical workflows.

#### 3.1.3. ^18^F-FET

^18^F-FET (O-(2-[^18^F]fluoroethyl)-L-tyrosine) is an amino acid-based PET tracer taken up by tumor cells via LAT1 transporters, mirroring L-tyrosine transport into rapidly proliferating cells [104]. Its low background uptake in normal brain tissue and high specificity for tumor regions make it well-suited for CNS imaging, particularly in gliomas [105,106]. Unlike MRI, which may struggle to differentiate tumors from post-surgical changes or recurrence, ^18^F-FET PET provides metabolic insights, improving diagnostic accuracy and treatment planning [107,108].

Studies confirm its utility in pediatric neuro-oncology. When added to MRI, ^18^F-FET PET improved diagnostic specificity (1.00 vs. 0.48) and overall accuracy in differentiating tumor from non-tumor tissue [49]. In a subset of patients, ^18^F-FET uptake was found to correlate with the genomic proliferation index, suggesting its potential role in molecular tumor characterization and personalized treatment strategies. A retrospective study further confirmed the high diagnostic accuracy of ^18^F-FET PET, reporting 100% sensitivity and 60% specificity for detecting recurrent or persistent pediatric CNS tumors while also accurately classifying all newly diagnosed tumors [50].

Clinically, ^18^F-FET PET was shown to influence management decisions in over two-thirds of pediatric patients by guiding biopsies, modifying treatment, or avoiding unnecessary interventions. It also enhanced diagnostic confidence in 67% and altered treatment plans in 87% of patients, including patients with DMG, anaplastic astrocytoma, and pilocytic astrocytoma (PA) [50,51].

^18^F-FET PET also plays a vital role in postoperative assessment, helping differentiate progression from treatment-related changes, with superior specificity compared to MRI (1.00 vs. 0.75), influencing therapy in 41% of patients [52]. In another study, it reliably identified residual tumor versus post-treatment effects in 94% of patients with various tumor types, including AP, MB, and glioblastoma multiforme (GBM) [53,54].

Finally, recent innovations in automated PET analysis further enhance the clinical utility of ^18^F-FET PET. Similar to improvements seen with ^18^F-DOPA PET, deep learning-based methods have demonstrated high accuracy in pediatric glioma delineation and a strong correlation between manual and AI-extracted clinical metrics [55]. These developments reduce inter-reader variability, improve the reproducibility of clinical assessments, and are expected to make ^18^F-FET PET an even more standardized and accessible tool for routine clinical use.

However, challenges remain. ^18^F-FET is less widely available than FDG or DOPA, and its relatively low affinity for LAT1 transporters may reduce uptake in some tumors [109,110,111]. To address this, new analogs, such as the meta-substituted ^18^F-FET (m-^18^F-FET), have demonstrated improved tumor accumulation in preclinical GBM models, making them a promising alternative for brain tumor imaging [112].

Overall, ^18^F-FET PET provides robust diagnostic, prognostic, and therapeutic insights in pediatric CNS tumors, with its role in personalized treatment planning and long-term monitoring continuing to grow, especially as multimodal imaging techniques evolve.

#### 3.1.4. ^18^F-Cho

^18^F-Cho, including ^18^F-fluorocholine and ^18^F-fluoroethylcholine, reflects membrane turnover and cell proliferation. Choline enters cells via choline transporters and is phosphorylated, a process that facilitates its incorporation into phospholipids for cell membrane synthesis—this is often upregulated in cancer cells [113]. These tracers are more established in adult oncology, especially for prostate cancer, but emerging data supports their utility in pediatric brain tumor imaging [114,115,116].

Initial investigations have demonstrated that simultaneous ^18^F-Cho PET and MRI are both feasible and clinically informative in pediatric astrocytic tumors, with tracer uptake aligning with contrast enhancement and restricted diffusion [56]. Reductions in SUVmax and SUVmean post-treatment were associated with tumor shrinkage, indicating response to therapy.

Further comparisons with advanced MRI techniques, including MRI spectroscopy and amide proton transfer–chemical exchange saturation transfer (APT-CEST), showed a strong correlation between APT-CEST signals and ^18^F-Cho SUV in teenage and young adult (TYA) gliomas, suggesting both modalities reflect proliferative activity and metabolic changes within the tumor [57]. Importantly, MRI spectroscopy techniques like APT-CEST can detect non-enhancing or infiltrative tumor regions not captured by ^18^F-Cho uptake, emphasizing the complementary value of combining PET and advanced MRI for a more comprehensive tumor assessment.

In a recent case series, ^18^F-Cho PET/MRI demonstrated utility across a spectrum of pediatric and TYA brain tumors [45]. For HGG, ^18^F-Cho uptake correlated with contrast enhancement and guided biopsy targeting, with follow-up scans showing complete metabolic response or confirming non-viable tumor despite residual MRI enhancement. In contrast, low-grade tumors showed more variable uptake. For example, a patient with MB exhibited no ^18^F-Cho uptake, underscoring limitations in detecting certain molecular subtypes. Another patient with a non-germinomatous germ cell tumor (NGGCT) exhibited moderate ^18^F-Cho uptake, corresponding to viable tumor at resection.

Altogether, these findings support ^18^F-Cho PET/MRI as a valuable tool for diagnosis, treatment monitoring, and intervention planning in pediatric and TYA brain tumors, especially for high-grade tumors. However, the variability of FCho uptake in certain tumor types highlights the importance of integrating ^18^F-Cho PET results with other imaging modalities and clinical data for accurate assessment.

#### 3.1.5. ^18^F-mFBG and ^124^I-mIBG

To address the previously mentioned drawbacks of ^123^I-mIBG scintigraphy in the management of NB, positron-emitting analogues of mIBG, such as ^18^F-mFBG (^18^F-meta-Fluorobenzyl-Guanidine) and ^124^I-mIBG, have been developed. ^18^F-mFBG offers higher sensitivity, quantitative imaging, and same-day acquisition, whereas ^124^I-mIBG preserves the same targeting mechanism but enables high-resolution PET/CT imaging [117]. These novel agents aim to enhance lesion detection and support more precise clinical decision-making in the management of pediatric NB.

Early clinical studies have confirmed the feasibility, safety, and favorable biodistribution of ^18^F-mFBG. In the first-in-human trial, skeletal and soft tissue lesions were visualized with high contrast, and no adverse events were reported [58]. The pre- and post-injection safety was further evaluated during an ongoing Phase I/II (NCT02348749) in pediatric NB, where no significant adverse safety signals were noted [59,60].

In comparative studies, ^18^F-mFBG PET/CT consistently detected more lesions than ^123^I-mIBG SPECT/CT. One prospective pilot study in children with stage 4 NB showed that ^18^F-mFBG identified more soft tissue lesions in 40% of paired scans [61]. Another phase II trial found that ^18^F-mFBG visualized 367 lesions versus 217 by ^123^I-mIBG, with follow-up confirming the clinical relevance of most discordant findings [62]. A larger prospective study further supported these findings, reporting that ^18^F-mFBG PET/CT detected significantly more lesions (784 vs. 532) and revealed 11.8% of cases that were false-negative on ^123^I-mIBG, reinforcing its superior sensitivity [63].

From a logistical perspective, ^18^F-mFBG PET/CT offers notable advantages. Long-axial-field-of-view (LAFOV) PET/CT enabled high-quality imaging within minutes—often eliminating the need for sedation, even in children under two years old [64].

Similarly, ^124^I-mIBG has shown enhanced lesion detection in a small prospective study of relapsed NB patients. In relapsed pediatric NB patients, PET/CT with ^124^I-mIBG visualized 87 lesions versus 25 and 32 by ^123^I-mIBG planar and SPECT/CT imaging, respectively [66]. Importantly, it offered improved anatomical coverage (chest, spine, extremities) and enabled whole-body PET/CT at a lower administered activity, though with a slightly higher effective dose due to its longer half-life.

Collectively, ^18^F-mFBG and ^124^I-mIBG represent promising PET-based alternatives to ^123^I-mIBG for NB imaging. Their superior sensitivity, improved workflow, and enhanced tolerability support their integration into clinical practice, pending further validation from larger multicenter trials, including a Phase III trial of ^18^F-mFBG (NCT04724369) [65].

#### 3.1.6. Additional Metabolism-Based Radiotracers

Beyond the tracers mentioned earlier, several additional metabolism-based radiotracers have been explored in pediatric brain tumors. These include ^18^F-FLT (^18^F-fluorothymidine), ^64^CuCl_2_, and 1-L-^18^F-FETrp (1-L-^18^F-fluoroethyl-tryptophan). Although most have been studied in limited preclinical or early clinical settings, they hold promise for broadening the molecular imaging toolkit in pediatric neuro-oncology.

^18^F-FLT is a thymidine analog that serves as a marker of cellular proliferation by reflecting thymidine kinase-1 activity [118]. In a multi-institutional study of 22 pediatric patients with newly diagnosed or recurrent brain tumors, ^18^F-FLT uptake strongly correlated with the histological Ki-67 labeling index, supporting its role as a non-invasive marker of tumor proliferative activity [67]. Importantly, in all cases with suspected recurrence, FLT PET correctly identified viable tumor, suggesting its utility in both diagnosis and recurrence assessment. This clinical study is followed by a larger Phase II trial (NCT01244737), aimed at validating ^18^F-FLT in pediatric CNS tumors for tumor grading, recurrence detection, and early therapy response monitoring [68].

^64^CuCl_2_ is a copper-based radiotracer that exploits altered copper metabolism in tumor cells, particularly via overexpression of copper transporters like CTR1, which support tumor development [119,120]. In a prospective study of 10 children with HGG (including diffuse midline and hemispheric gliomas), ^64^CuCl_2_ PET demonstrated selective uptake in enhancing tumor regions, particularly those showing necrosis [69]. Uptake increased over 1 to 72 h post-injection and aligned with blood-brain barrier disruption. These findings suggest both diagnostic and potential theranostic applications of copper-based tracers in pediatric glioma.

Finally, 1-L-^18^F-FETrp, a novel tryptophan derivative targeting the kynurenine pathway, was evaluated in a transgenic mouse model of MB [70,71,121]. PET imaging showed high and specific tumor uptake, with low background in normal brain tissue, outperforming ^18^F-FDG. This highlights its potential as a selective imaging probe for MB and possibly other pediatric brain tumors.

Collectively, these emerging metabolic radiotracers expand the molecular imaging toolbox for pediatric brain tumors, offering new avenues for non-invasive tumor characterization, proliferation assessment, and treatment monitoring. Further validation in larger, prospective clinical trials will be critical to define their roles in routine pediatric neuro-oncology practice.

### 3.2. Molecular Imaging and Targeted Radiotherapy with Peptide Receptor-Based Radiotracers

#### 3.2.1. ^68^Ga- and ^177^Lu-DOTATATE

DOTATATE-based (DOTA-Tyr3-Octreotate) radiotracers, including ^68^Ga-DOTATATE for PET imaging and ^177^Lu-DOTATATE for peptide receptor radionuclide therapy (PRRT), selectively target somatostatin receptor subtype 2 (SSTR2), highly expressed in neuroendocrine tumors (NETs) [122]. Both agents have received regulatory approval for use in adult and pediatric SSTR2-positive NETs [123,124] and are now being explored in NB, particularly in cases refractory to mIBG-based imaging and therapy [28,125]. Given their potential to improve both imaging accuracy and therapeutic outcomes, DOTATATE-based approaches hold promising clinical value, especially in advanced or refractory NB.

Recent studies underscore the diagnostic superiority of ^68^Ga-DOTATATE PET in pediatric NB, especially in staging, restaging, and evaluation of treatment response. Compared to ^123^I-mIBG SPECT, ^68^Ga-DOTATATE PET showed higher sensitivity and spatial resolution, with improved detection of bone marrow involvement and early progression [72]. In one series, all patients showed ^68^Ga-DOTATATE-avid disease, while ^123^I-mIBG was negative in two cases and partially discordant in others, supporting their complementary roles [73]. Finally, several reports describe cases where ^68^Ga-DOTATATE/CT identified intracranial or spinal dural metastases that were missed by ^123^I-mIBG SPECT/CT, underscoring its potential in evaluating CNS involvement [74,75]. These findings suggest that ^68^Ga-DOTATATE may improve lesion localization and could help guide patient selection for ^177^Lu-DOTATATE therapy, particularly in patients with heterogeneous expression of somatostatin receptors.

The therapeutic counterpart, ^177^Lu-DOTATATE, has been evaluated in NB patients guided by 68Ga-DOTATATE imaging. In one study, PRRT induced objective responses in refractory cases and was well tolerated [76]. Another trial combining ^177^Lu-DOTATATE with chemotherapy reported partial or complete responses in 3 of 5 treated children with manageable hematologic toxicity [77]. These findings suggest that PRRT, guided by ^68^Ga-DOTATATE imaging, may be an effective salvage strategy in mIBG-refractory disease. Moreover, a recent case report highlighted the utility of ^68^Ga-DOTATATE PET/CT in identifying residual bone metastases undetected by ^123^I-mIBG following ^177^Lu-DOTATATE PRRT, reinforcing its value in post-treatment assessment [78].

Despite encouraging results from case series and small trials, a phase IIa clinical trial evaluating ^177^Lu-DOTATATE monotherapy (LuDO) in children with relapsed/refractory NB did not demonstrate objective responses, though treatment was generally well tolerated [79]. Early pharmacokinetic analyses from the LuDO trial revealed decreasing tumor absorbed doses across treatment cycles, suggesting the need for adaptive dosimetry to maintain efficacy [80]. This outcome prompted the development of the ongoing LuDO-N trial, which utilizes a personalized, dose-intense administration schedule to optimize therapeutic efficacy of ^177^Lu-DOTATATE in children with high-risk NB [81].

Taken together, these studies indicate that while ^177^Lu-DOTATATE monotherapy may have limited standalone activity in high-risk NB, its integration with diagnostic ^68^Ga-DOTATATE PET, combination regimens, and individualized dosimetry holds potential for improved outcomes in selected patients—namely those with SSTR-positive, relapsed, or refractory disease eligible for PRRT.

#### 3.2.2. ^68^Ga-DOTANOC

While DOTATATE-based radiotracers targeting SSTR2 have shown promise in NB management, recent evidence highlights SSTR2A expression in several pediatric CNS tumors. A large immunohistochemical study found particularly high expression in MB and meningioma, suggesting potential utility for SSTR-targeted imaging and therapy beyond extracranial neuroendocrine tumors [126].

^68^Ga-DOTANOC (^68^Ga-DOTA-NaI3-Octreotide), a PET tracer with broader affinity for SSTR2, SSTR3, and SSTR5, has been evaluated in pediatric CNS tumors [127]. Two case reports describe its use in children with metastatic MB for treatment response assessment [82,83]. In both cases, ^68^Ga-DOTANOC PET/CT successfully identified extensive skeletal metastases at baseline and showed complete resolution of tracer uptake following chemotherapy, paralleling findings on ^18^F-FDG PET/CT.

Although data remain limited, these preliminary findings support the feasibility of SSTR-targeted PET imaging in pediatric CNS tumors. Given the SSTR expression profile, particularly in MB, tracers such as ^68^Ga-DOTANOC warrant further investigation for diagnostic and potentially therapeutic applications in pediatric neuro-oncology.

#### 3.2.3. Additional Peptide Receptor-Based Radiotracers

In addition to established receptor-targeted tracers, several novel radiotracers are being investigated for their potential in pediatric brain tumor imaging. These agents target distinct molecular markers, such as gastrin-releasing peptide receptor (GRPR), translocator protein (TSPO), and integrin-αvβ3, that have been shown to be overexpressed in various CNS tumors [128,129,130,131,132]. Although many of these approaches remain in early clinical or preclinical settings, they highlight promising directions for expanding molecular imaging capabilities in pediatric neuro-oncology.

^68^Ga-NOTA-Aca-BBN (7-14), a GRPR-targeting PET tracer derived from bombesin (BBN), was evaluated in a prospective study involving eight children with suspected optic pathway glioma (OPG) [84]. All 11 lesions across patients showed high tracer uptake with excellent tumor-to-background contrast, markedly outperforming ^18^F-FDG PET/CT. GRPR expression was confirmed histopathologically in all cases, and uptake correlated with receptor density. PET/MRI fusion imaging further aided in tumor delineation and surgical navigation, demonstrating the feasibility and potential clinical utility of GRPR-targeted imaging in this setting.

^18^F-DPA-714, a TSPO-targeted PET tracer, has been studied in a patient-derived orthotopic xenograft rat model of DMG [85]. Kinetic modeling and parametric imaging enabled clear delineation of tumor extent, overcoming some of the limitations of conventional MRI. Given the inaccessibility of DMG for biopsy and the need for non-invasive biomarkers, TSPO imaging may offer a valuable tool for tumor monitoring and radiotherapy planning.

Finally, integrin-αvβ3 expression has been identified as a marker of tumorigenicity and radioresistance in MB [86,133]. Preclinical studies using ^99m^Tc-RAFT-RGD SPECT/MRI demonstrated successful visualization of integrin-positive tumors in orthotopic mouse models. Notably, integrin-αvβ3 expression increased in radioresistant cell lines and was amenable to pharmacologic inhibition, suggesting both diagnostic and therapeutic relevance in a subset of patients.

Collectively, these emerging tracers targeting GRPR, TSPO, and integrin-αvβ3 demonstrate feasibility for receptor-based imaging in pediatric CNS tumors. While still in early development, they offer compelling avenues for improving diagnostic precision, guiding treatment planning, and enabling future theranostic applications in pediatric neuro-oncology.

### 3.3. Molecular Imaging and Targeted Radiotherapy with Antibody-Based Radiotracers

#### 3.3.1. ^124^I- and ^131^I-Omburtamab

Monoclonal antibodies are increasingly explored as theranostic agents in pediatric neuro-oncology, offering tumor-specific targeting for both imaging and therapy [134]. Among them, ^124^I- and ^131^I-omburtamab (previously named 8H9), a radiolabeled antibody against B7-H3 (CD276), have shown promise for PET-based dosimetry and targeted treatment in refractory CNS tumors. B7-H3 is an immune checkpoint molecule overexpressed in tumors such as DMG and MB and is linked to immune evasion and tumor aggressiveness, making it a compelling therapeutic target [135].

Early preclinical studies demonstrated the safety and feasibility of convection-enhanced delivery (CED) of ^124^I-omburtamab to the brainstem in rodent and primate models. These studies highlighted high T/B ratios and minimal systemic exposure, providing promising preclinical data for clinical translation [136].

Following these findings, clinical trials were conducted in pediatric DMG. A Phase I trial demonstrated the safety of CED for ^124^I-omburtamab delivery directly to the brainstem in children with DMG who have previously received radiation therapy, emphasizing its feasibility for targeting hard-to-reach tumors in the brainstem with high intra-lesional dosing [87]. Another study also demonstrated the efficacy of ^124^I-omburtamab PET in DMG patients with the use of CED. Tumor lesions exhibited favorable T/B ratios with prolonged lesion retention and minimal systemic distribution, confirming its potential for PET-based dosimetry [88]. A subsequent Phase I dose-escalation trial involving 50 children with DMG treated with escalating doses of ^124^I-omburtamab after radiotherapy found that 6 mCi was the maximum tolerated dose. The study demonstrated a high lesion-to-whole-body absorbed dose ratio and a median overall survival of 15.3 months, which exceeded typical expectations for this population [89].

^124^I-omburtamab has also been evaluated in pediatric patients with leptomeningeal disease, primarily those with NB. Intraventricular administration of ^124^I-omburtamab for PET imaging allowed accurate lesion dosimetry and revealed high cerebrospinal fluid (CSF)-to-blood absorbed dose ratios, which were essential for guiding subsequent therapy with ^131^I-omburtamab [90].

Furthermore, a study in 95 patients, primarily with NB, MB, and ependymoma, confirmed that pretreatment imaging with intraventricular ^131^I-omburtamab itself using serial planar SPECT imaging can also provide detailed dosimetric data [91]. This approach allowed precise dosimetric measurements for ventricular and spinal CSF compartments, with rapid localization to target regions and minimal systemic distribution. The dosimetric data from the ^131^I-omburtamab imaging closely aligned with previously reported ^124^I-omburtamab PET data, confirming its feasibility in clinical practice.

Moreover, the use of ^131^I-omburtamab for intraventricular compartmental radioimmunotherapy (cRIT) has been evaluated in a Phase I dose-escalating trial, which included children with CNS malignancies (NCT00089245). The results showed that the treatment was well-tolerated, even at high doses, with mild headaches, vomiting, and thrombocytopenia as the most common toxicities. cRIT demonstrated a marked survival benefit in NB patients, with a median PFS of 7.5 years. For MB and ependymoma patients, PFS and OS were significantly improved in those with no evidence of disease at treatment initiation, defined by MRI and clinical assessment, with 11 of 20 medulloblastoma and 3 of 7 ependymoma patients classified as NED in the trial [92]. Importantly, there were no reports of radionecrosis, which supports the favorable safety profile of this approach in the pediatric population. While radionecrosis and pseudoprogression can be markers of therapeutic response in adults, their absence here may reflect the controlled, compartmentalized delivery of ^131^I-omburtamab. Additionally, ^131^I-omburtamab was well-tolerated in three pediatric patients with embryonal tumors with multilayered rosettes (ETMR), delivering high radiation doses to tumors while minimizing exposure to blood and CSF [93]. Two patients remained in remission for 2.3 and 6.8 years, while one patient succumbed to disease progression 7 months after therapy. These findings suggest that ^131^I-omburtamab may offer therapeutic benefit for ETMR patients, particularly when used as consolidation following surgery and chemoradiation therapy.

These studies collectively support ^124^I-omburtamab and ^131^I-omburtamab as promising theranostic agents for pediatric brain tumors, offering enhanced diagnostic accuracy and therapeutic potential, particularly in difficult-to-treat conditions like DMG and leptomeningeal disease.

#### 3.3.2. ^89^Zr-Dinutuximab and ^64^Cu-Dinutuximab-Beta

GD2, a disialoganglioside highly expressed on NB and other pediatric tumors, has emerged as a critical target for immunotherapy due to its restricted expression in normal tissues and its involvement in tumor progression [137]. The FDA-approved anti-GD2 monoclonal antibody dinutuximab (ch14.18; Unituxin^®^) has demonstrated significant efficacy in treating relapsed and refractory NB, while dinutuximab beta (ch14.18/CHO; Qarziba^®^), approved by the EMA, is now the standard care for high-risk NB patients with partial responses to prior treatments [138,139,140]. Ongoing clinical trials are evaluating the use of these antibodies in other GD2-positive pediatric tumors, such as osteosarcoma (NCT02484443), leiomyosarcoma (NCT05080790), and Ewing’s sarcoma [141,142,143]. As GD2-targeted therapies continue to expand, the development of molecular imaging strategies to assess GD2 expression and monitor treatment responses is essential.

A major advancement in GD2-targeted imaging is the development of radiolabeled agents like ^89^Zr-dinutuximab, a PET tracer designed to bind GD2-expressing tumors. Early preclinical studies have shown high specificity for NB tumors with excellent T/B ratios, suggesting its potential as an effective diagnostic tool for evaluating GD2 expression prior to therapy [94]. Building on this success, dinutuximab beta was radiolabeled with ^64^Cu for PET imaging. In vivo studies confirmed that the radiotracer selectively binds to GD2-positive NB tumors with minimal off-target activity [95]. This was followed by the first clinical application of ^64^Cu-dinutuximab beta PET/MRI in a NB patient, where metastatic lesions were successfully visualized, demonstrating the technique’s diagnostic potential.

Furthermore, a recent clinical study with ^64^Cu-dinutuximab beta PET/MRI in 11 pediatric patients, including 6 with NB, demonstrated its clinical relevance [144]. Of the 6 NB patients, 5 showed detectable tumor uptake, with 2 exhibiting high uptake and 3 moderate to low uptake. In one patient, intense radiotracer uptake in bone metastases contradicted GD2-negative histology in a dura metastasis, highlighting the heterogeneity of GD2 expression in NB. These findings reinforce the utility of PET imaging for detecting GD2 expression, even in cases where histology may fail. Additionally, the low radiation dose associated with ^64^Cu-dinutuximab beta further supports its feasibility as a clinical imaging tool.

In conclusion, GD2-targeted PET imaging, particularly with ^64^Cu-dinutuximab beta, shows promise as a non-invasive tool for assessing GD2 expression in pediatric cancers. This approach can help optimize treatment strategies by identifying suitable candidates as well as monitoring therapeutic responses in real-time. As clinical applications grow, further validation in larger cohorts and across different GD2-positive tumors is crucial for routine clinical use.

#### 3.3.3. ^89^Zr-Bevacizumab

Bevacizumab (Avastin^®^) is a monoclonal antibody targeting vascular endothelial growth factor (VEGF), a protein that promotes tumor angiogenesis, thereby supporting tumor growth [145]. While bevacizumab is FDA-approved for second-line treatment of glioblastoma multiforme in adults, its role in pediatric brain cancers is still being evaluated [146,147,148,149]. It has been assessed mostly as an adjunctive treatment for pediatric brain tumors, with varying degrees of success—especially in DMG [150,151,152,153]. This variability underscores the need for non-invasive molecular imaging tools to better predict which patients are likely to benefit from therapy.

^89^Zr-labeled bevacizumab PET imaging has been utilized to evaluate the biodistribution and tumor uptake of bevacizumab in both preclinical and clinical settings. In mouse models of DMG, the effectiveness of ^89^Zr-bevacizumab in targeting tumors was found to depend on VEGF expression levels and the presence of vascular proliferation [154]. However, in DMG xenografts located in the pons and striatum, no significant uptake was observed, indicating limited efficacy in the diffuse, infiltrative parts of the brain.

Clinical ^89^Zr-bevacizumab PET imaging in children with DMG demonstrated feasibility and revealed important insights into the heterogeneity of drug delivery within tumors. In a study of seven DMG patients, five showed focal uptake of ^89^Zr-bevacizumab, primarily in MRI contrast-enhanced areas, suggesting a positive correlation between VEGF-targeted imaging and MRI findings [96]. Furthermore, ^89^Zr-bevacizumab PET revealed substantial intra- and intertumoral heterogeneity, highlighting the need for personalized treatment strategies based on these imaging results.

In a more detailed analysis, a study combining ^89^Zr-bevacizumab PET imaging with histological evaluation of lesions from a DMG patient found heterogeneous tracer uptake across different lesions. Increased uptake correlated with microvascular proliferation, but not necessarily with VEGF expression levels, indicating that other factors influence the uptake heterogeneity [97]. This study underscored the importance of optimized timing and patient selection when considering therapies targeting the complex and heterogeneous DMG microenvironment.

In conclusion, ^89^Zr-bevacizumab PET imaging shows promise for enhancing patient selection and monitoring therapeutic efficacy in pediatric brain tumors, especially with VEGF-targeted therapies like bevacizumab. Further research and optimization of imaging techniques are essential for developing more personalized and effective treatments for DMG and other pediatric brain cancers.

## 4. Conclusions

The integration of advanced molecular imaging techniques—including metabolic, receptor-based, and antibody-targeted PET tracers—is revolutionizing pediatric neuro-oncology. These approaches enable more precise tumor characterization, enhanced treatment monitoring, and the development of personalized therapeutic strategies, particularly for complex tumors like DMG. By targeting specific biomarkers such as B7-H3, GD2, and VEGF, these tracers also support theranostic applications that combine diagnosis and therapy, as seen with radiolabeled antibodies like ^124^I/^131^I-omburtamab and ^64^Cu/^89^Zr-dinutuximab. However, it is important to note that most available clinical studies involve small pediatric cohorts, often with fewer than ten patients, limiting the statistical strength and generalizability of the findings. While further research is essential to refine imaging protocols and expand their clinical use, larger multicentric or retrospective studies are needed to validate preliminary results and establish clear recommendations for clinical practice. Additionally, while most current studies rely on static imaging and SUV quantification, kinetic analyses such as time–activity curve evaluation and compartmental modeling may offer deeper insight into tracer behavior and tumor biology. Incorporating such techniques in future pediatric studies could improve diagnostic accuracy and enhance therapy response assessment. The potential to significantly improve diagnostic precision and patient outcomes is increasingly evident. The continued advancement and clinical integration of these technologies will be instrumental in shaping the future of pediatric brain tumor management through more individualized and effective care.

## Abbreviations

APT-CEST	Amide proton transfer–chemical exchange saturation transfer
ASL	Arterial spin labeling
GBM	Glioblastoma Multiforme
CED	Convection-Enhanced Delivery
CSF	Cerebrospinal fluid
CNS	Central Nervous System
CRIT	Combined Radioimmunotherapy
CT	Computed Tomography
cRIT	Compartmental Radioimmunotherapy
DAT	Diffuse astrocytic tumors
DIPG	Diffuse Intrinsic Pontine Glioma
DMP	Diffuse Midline Glioma with H3K27M Mutation
DWI	Diffusion-weighted imaging
EMA	European Medicines Agency
ETMR	Embryonal Tumor with Multilayered Rosettes
FDG	Fluorodeoxyglucose
FET	Fluoroethyl-L-tyrosine
HGG	High-Grade Glioma
LGG	Low-Grade Glioma
LAFOV	Long-axial-field-of-view
MB	Medulloblastoma
mIBG	Meta-Iodobenzylguanidine
MRI	Magnetic Resonance Imaging
MTV	Metabolic Tumor Volume
NB	Neuroblastoma
NETs	Neuroendocrine Tumors
NGGCT	Non-Germinomatous Germ Cell Tumor
OPG	Optic Pathway Glioma
OS	Overall Survival
PET	Positron Emission Tomography
PFS	Progression-Free Survival
SPECT	Single Photon Emission Computed Tomography
SSTR2	Somatostatin Receptor Type 2
TYA	Teen and Young Adult
VEGF	Vascular Endothelial Growth Factor.
